# Long‐term efficacy of eculizumab in refractory generalized myasthenia gravis: responder analyses

**DOI:** 10.1002/acn3.51376

**Published:** 2021-05-27

**Authors:** James F. Howard, Chafic Karam, Marcus Yountz, Fanny L. O’Brien, Tahseen Mozaffar, for the REGAIN Study Group

**Affiliations:** ^1^ Department of Neurology The University of North Carolina Chapel Hill North Carolina USA; ^2^ Department of Neurology Oregon Health & Science University Portland Oregon USA; ^3^ Alexion Pharmaceuticals, Inc. Boston Massachusetts USA; ^4^ Department of Neurology University of California Irvine California USA; ^5^ Present address: Penn Neuroscience Center ‐ Neurology, Hospital of the University of Pennsylvania Philadelphia Pennsylvania USA

## Abstract

**Objective:**

Generalized myasthenia gravis (gMG) is an autoimmune disease that causes disabling weakness via damage to the neuromuscular junction. In most patients, the disease is mediated by autoantibodies to the acetylcholine receptor, which activate the complement cascade. Our objective was to analyze response profiles in adult patients with anti‐acetylcholine receptor antibody‐positive refractory gMG treated with eculizumab—a terminal complement inhibitor—in the REGAIN study or its open‐label extension (OLE).

**Methods:**

We retrospectively analyzed Myasthenia Gravis‐Activities of Daily Living (MG‐ADL) and Quantitative Myasthenia Gravis (QMG) scores recorded during REGAIN and its OLE. Early/late responses were defined as improvement in MG‐ADL score (≥3 points) or QMG score (≥5 points) at ≤12 or >12 weeks, respectively, after eculizumab initiation.

**Results:**

The analysis included 98 patients. By Week 12 and conclusion of the OLE, MG‐ADL response had been achieved at some point by 67.3% and 84.7% of patients, respectively, and QMG response by 56.1% and 71.4%, respectively. Response was observed over multiple consecutive assessments for most patients. At Week 130, the least‐squares mean percentage changes (95% CI) from baseline in MG‐ADL score were −61.9% (−69.9%, −53.9%) and −47.5% (−59.0%, −36.0%) in early and late MG‐ADL responders, respectively; the least‐squares mean percentage changes from baseline in QMG score were −40.8% (−48.3%, −33.4%) and −55.5% (−68.4%, −42.7%) in early and late QMG responders, respectively.

**Interpretation:**

The findings suggest that, although most patients with refractory gMG will achieve clinical response by Week 12 of eculizumab treatment, first responses can be observed with longer‐term treatment.

## Introduction

Generalized myasthenia gravis (gMG) is a prototypical autoimmune disease resulting from antibody‐mediated damage of the neuromuscular junction.^1^, ^2^ The majority (~85%) of patients with the disease have antibodies against the acetylcholine receptor (AChR),^3^ which cause pathogenic effects at the postsynaptic membrane of the neuromuscular junction via several processes, primarily complement‐mediated membrane damage.^4^, ^5^, ^6^, ^7^, ^8^, ^9^, ^10^

Treatments for gMG include acetylcholinesterase inhibitors, corticosteroids, and steroid‐sparing immunosuppressive therapies (ISTs). However, 10–15% of patients do not respond adequately or are unable to tolerate ISTs, and are considered treatment refractory.^11^, ^12^ Patients who are treatment refractory continue to have debilitating symptoms and persistent morbidities, and experience frequent exacerbations, hospitalizations, and myasthenic crises that can be life‐threatening.^11^, ^13^

Eculizumab (Soliris^®^, Alexion Pharmaceuticals, Boston, MA, USA) is a humanized monoclonal antibody that specifically binds with high affinity to human terminal complement protein C5, thereby inhibiting C5 cleavage to proinflammatory complement complexes.^14^ The efficacy and tolerability of eculizumab in patients with anti‐AChR‐positive refractory gMG were demonstrated in the 26‐week REGAIN study, in which participants treated with eculizumab experienced clinically meaningful benefits in activities of daily living, muscle strength, functional ability, and quality of life.^15^ The long‐term safety and sustained efficacy of eculizumab were subsequently shown in the REGAIN open‐label extension (OLE) trial.^16^

Here we report a retrospective analysis of responder data for patients enrolled in the REGAIN trial and its OLE. The primary research question was to determine the timing of clinical response in adult patients with anti‐AChR antibody‐positive refractory gMG treated with eculizumab. Baseline/demographic factors that may predict the likelihood of a response were also examined.

## Materials and Methods

### REGAIN and its open‐label extension

Full details of the methodology for REGAIN and its OLE study have been reported previously.^15^, ^16^ In brief, the REGAIN study included patients aged ≥18 years with confirmed gMG, a positive serological test for anti‐AChR antibodies, impaired activities of daily living (i.e., Myasthenia Gravis‐Activities of Daily Living [MG‐ADL] score of ≥6), and disease class II–IV according to the Myasthenia Gravis Foundation of America (MGFA). Patients were required to have received at least two ISTs for 12 months without symptom control or at least one IST plus intravenous immunoglobulin (IVIg) or plasma exchange (PLEX) administered at least four times per year for 12 months without symptom control (i.e., treatment refractory).

During REGAIN, patients were randomized to intravenous eculizumab or placebo for 26 weeks. Eculizumab dosing was 900 mg on Day 1 and at Weeks 1, 2, and 3; 1200 mg at Week 4; and 1200 mg every second week thereafter as maintenance dosing. Those receiving prior therapy with a cholinesterase inhibitor, oral corticosteroid, or other ISTs continued that treatment at the same dose and schedule throughout the study. Patients who completed REGAIN could enter into the OLE study (up to 208 weeks), in which all patients received eculizumab maintenance therapy (1200 mg every 2 weeks). To preserve the blinded nature of REGAIN, patients who entered the OLE first underwent a 4‐week blinded induction phase during which investigators, patients, and study personnel remained blinded to all treatment assignments. During this phase, patients who had been assigned to eculizumab in REGAIN received eculizumab 1200 mg (four vials) on Day 1 and Week 2, and placebo (four vials) at Weeks 1 and 3. Patients who had been assigned to placebo in REGAIN received eculizumab (900 mg, three vials) and placebo (one vial) on Day 1 and at Weeks 1, 2, and 3. During the OLE, the dose of concomitant ISTs could be modified at the investigator’s discretion. Rescue medication (e.g., high‐dose corticosteroids, IVIg, or PLEX) was permitted at the physician’s discretion during REGAIN and its OLE.

Efficacy was assessed using the patient‐reported MG‐ADL score and the clinician‐administered Quantitative Myasthenia Gravis (QMG) scale, with assessments performed weekly from Week 1 through Week 4, at Weeks 8, 12, 16, 20, and 26, or at early termination in REGAIN, and from Week 1 through Week 4, at Weeks 8, 12, 16, 20, 26, 40, and 52 in Year 1, every 6 months thereafter, and at each patient’s end‐of‐study visit in the OLE. To assess safety, the incidence of adverse events and other safety measures were recorded.

### Responder analysis

In this retrospective analysis, the response profiles of participants in REGAIN and its OLE were explored according to MG‐ADL and QMG scores recorded during the studies. The MG‐ADL scale is a validated eight‐item outcome measure that reflects ocular, bulbar, respiratory, and limb symptoms and their impact on function.^17^ Each item is graded on a 4‐point symptom severity scale (where 0 = normal, 3 = most severe), with the total score ranging from 0 to 24; higher scores indicate greater functional impairment and disability. The QMG scale is a 13‐item scale that evaluates muscle strength based on the quantitative testing of sentinel muscle groups: ocular, facial, bulbar, gross motor, axial, and respiratory.^18^, ^19^ All items are scored on a scale of 0–3, and the total score ranges from 0 to 39; a higher score indicates greater disease severity.

In line with the REGAIN study and its OLE, response for the current analysis was defined as a ≥3‐point reduction in the MG‐ADL total score or a ≥5‐point reduction in the QMG total score from the start of eculizumab baseline. It should be noted that the magnitude of these response cut‐offs exceeds the minimal clinically important differences for each measure (≥2 points for MG‐ADL^20^ and ≥3 points for QMG^21^). Early responders were defined as patients in whom a response was seen on or before the Week 12 assessment; late responders were defined as those in whom a response was seen after Week 12 through to the final assessment in the OLE; non‐responders were defined as patients in whom no response was observed up to and including their last assessment in the OLE. For the classification of early and late responders, patients had to experience a response only once in the timeframe, rather than for a sustained period.

Analyses included the percentage of MG‐ADL/QMG early, late, and non‐responders, time to MG‐ADL/QMG response, and change (and percent change) from baseline in MG‐ADL/QMG total score over time for early and late responders. MG‐ADL/QMG response groups were analyzed to examine whether there were differences in demographics and baseline characteristics between the three response groups. Formal statistical comparisons were conducted for the variables: age at first eculizumab dose; sex; duration of myasthenia gravis (MG); baseline MG‐ADL/QMG score; MGFA class at screening; the number of ISTs used before REGAIN; and the number of patients with an MG crisis before REGAIN. Descriptive comparisons were conducted for further analyses of disease severity, including the number of prior exacerbations, the number of patients with 0, 1, 2, or 3+ prior MG crises, and history of thymectomy, and also to understand whether there were differences between the groups regarding the use of specific ISTs. An additional analysis was conducted to establish whether there were differences in the number of assessments conducted for each of the response groups.

### Statistical analysis

The response analysis population comprised all patients who received eculizumab during REGAIN and its OLE and who had an MG‐ADL total score of ≥6 at eculizumab initiation. Baseline for patients receiving eculizumab in the REGAIN study was the REGAIN baseline assessment. Baseline for those receiving placebo during REGAIN was the open‐label baseline assessment in the OLE study. Descriptive statistics are provided.

Differences in baseline demographics between two categories of responders were examined. For the MG‐ADL/QMG early‐ and late‐responder groups, percent changes from baseline over time were analyzed with mixed‐effect repeated measure models including terms of visit and baseline value.

### Measurement of terminal complement inhibition

The serum free C5 concentration is a pharmacodynamic measurement of terminal complement activity. Free C5 was quantified using a validated enzyme‐linked immunosorbent assay. The lower limit of quantification was 0.0274 μg/mL. A free C5 concentration of <0.5 µg/mL correlates with complete blockade of terminal complement activity in patients with refractory gMG.

### Standard protocol approvals, registrations, and patient consents

The protocols and all amendments for the studies from which the present analysis was derived (REGAIN [NCT01997229] and its OLE [NCT02301624]) were approved in writing by independent ethics committees or institutional review boards at all participating sites. All participants provided written informed consent.

## Results

After excluding patients who received placebo during REGAIN and whose MG‐ADL total score at the start of the OLE was <6, the response analysis population comprised 98 patients treated with eculizumab. Details of the patient populations for REGAIN, the REGAIN OLE, and the response analysis are shown in Figure S1.

### Response profiles

By Week 12 of eculizumab treatment, an MG‐ADL response had been achieved at some point by 66 patients (67.3%) (MG‐ADL early responders); a further 17 (17.3%) achieved an MG‐ADL response between Week 12 and the end of the OLE (MG‐ADL late responders); and 15 (15.3%) did not achieve the defined MG‐ADL response during eculizumab treatment (MG‐ADL non‐responders; Table 1). With regard to QMG, by Week 12, a response had been achieved at some point by 55 patients (56.1%) (QMG early responders); a further 15 patients (15.3%) had achieved a QMG response between Week 12 and the end of the OLE (QMG late responders); and 28 (28.6%) did not achieve the defined QMG response during eculizumab treatment (QMG non‐responders; Table 2). Of the 66 MG‐ADL early responders, 50 (75.8%) were also QMG early responders and of the 55 QMG early responders, 50 (90.9%) were also MG‐ADL early responders. All patients, regardless of the responder group, had rapid, complete, and sustained terminal complement inhibition, with no correlation between time to complete complement inhibition and time to MG‐ADL or QMG response (data not shown).

**Table 1:** Patients first achieving a response according to their Myasthenia Gravis‐Activities of Daily Living score, by assessment visit (response analysis set).

Visit assessment	Patients with a first response (*N* = 98), *n* (%)	Cumulative number of patients with a response (*N* = 98), *n* (%)
Week 1	32 (32.7)	32 (32.7)
Week 2	14 (14.3)	46 (46.9)
Week 3	5 (5.1)	51 (52.0)
Week 4	5 (5.1)	56 (57.1)
Week 8	7 (7.1)	63 (64.3)
Week 12	3 (3.1)	66 (67.3)
Week 16	2 (2.0)	68 (69.4)
Week 20	2 (2.0)	70 (71.4)
Week 26	2 (2.0)	72 (73.5)
After Week 26	11 (11.2)	83 (84.7)
No response	15 (15.3)	–

**Table 2:** Patients first achieving a Quantitative Myasthenia Gravis response by assessment visit (response analysis set).

Visit assessment	Patients with a first response (*N* = 98), *n* (%)	Cumulative number of patients with a response (*N* = 98), *n* (%)
Week 1	19 (19.4)	19 (19.4)
Week 2	13 (13.3)	32 (32.7)
Week 3	10 (10.2)	42 (42.9)
Week 4	4 (4.1)	46 (46.9)
Week 8	6 (6.1)	52 (53.1)
Week 12	3 (3.1)	55 (56.1)
Week 16	2 (2.0)	57 (58.2)
Week 20	4 (4.1)	61 (62.2)
Week 26	0	61 (62.2)
After Week 26	9 (9.2)	70 (71.4)
No response	28 (28.6)	–

**FIGURE 1:**
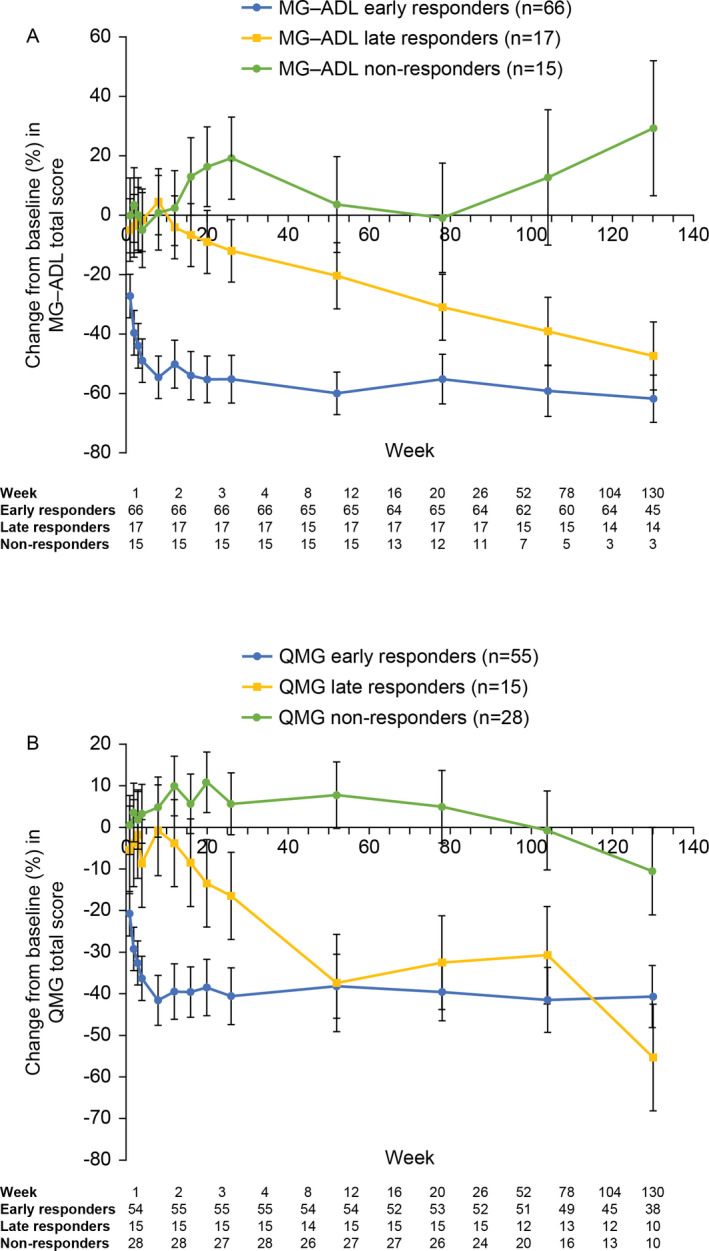
Efficacy of eculizumab (response analysis set). Percent change from baseline in (A) MG‐ADL total score and (B) QMG total score. Data are least‐squares mean ± 95% confidence interval estimates. MG‐ADL, Myasthenia Gravis‐Activities of Daily Living; QMG, Quantitative Myasthenia Gravis.

Among the early responders according to MG‐ADL score, significant improvement in the corresponding total score (least‐squares mean [LSM] percentage change from baseline −27.3%; 95% confidence interval [CI]: −34.6%, −20.0%) was observed by Week 1 (Fig. 1). Among late responders, a steady improvement in the total score was observed from Week 12, reaching statistical significance from Week 26 (Fig. 1). At Week 130, LSM percentage change from baseline was −61.9% (95% CI: −69.9%, −53.9%) and −47.5% (95% CI: −59.0%, −36.0%) for MG‐ADL early and late responders, respectively (Fig. 1). Thirty (45.5%) of the 66 MG‐ADL early responders were classified as MG‐ADL responders at all assessments including and following the timepoint at which the response was initially observed. The majority of MG‐ADL early responders (59/66; 89.4%) were classified as responders at >50% of assessments including and following the first response timepoint. Overall, for MG‐ADL early responders, response was recorded at 85.1% (941/1106) of assessments after the first response.

Of the 17 MG‐ADL late responders, four (23.5%) were classified as MG‐ADL responders at every assessment following the initial response; 10 (58.8%) were classified as MG‐ADL responders at >50% of timepoints including and following the first response. Overall, an MG‐ADL response was recorded at 58.5% (76/130) of assessments after the first response. Of the 47 early and late responders (58.0%) who did not maintain the 3‐point MG‐ADL response at every subsequent assessment, seven patients (14.9%) maintained at least a 2‐point response (Table 3). Further breakdown of the data for patients maintaining MG‐ADL response is shown in Table S1.

**Table 3:** Worst‐case status after the first response.

Patients, *n* (%)	MG‐ADL		QMG	
	Early responders (*n* = 66)	Late responders (*n* = 17)	Early responders (*n* = 55)	Late responders (*n* = 15)
Patients with follow‐up assessments after the first response	66 (100.0)	15 (88.2)	54 (98.2)	15 (100.0)
Patients maintaining response	30 (45.5)	4^a^ (23.5)	19^b^ (34.5)	1 (6.7)
Patients experiencing the following worst case after the first response:				
4‐point improvement from baseline	–	–	4 (7.3)	4 (26.7)
3‐point improvement from baseline	–	–	2 (3.6)	2 (13.3)
2‐point improvement from baseline	5 (7.6)	2 (11.8)	9 (16.4)	1 (6.7)
1‐point improvement from baseline	9 (13.6)	4 (23.5)	6 (10.9)	2 (13.3)
0‐point improvement (return to baseline)	8 (12.1)	3 (17.6)	3 (5.5)	2 (13.3)
1‐point worsening from baseline	5 (7.6)	1 (5.9)	4 (7.3)	0 (0.0)
2‐point worsening from baseline	6 (9.1)	1 (5.9)	1 (1.8)	1 (6.7)
3‐point worsening from baseline	1 (1.5)	0 (0.0)	1 (1.8)	0 (0.0)
≥4‐point worsening from baseline	2 (3.0)	0 (0.0)	5 (9.1)	2 (13.3)

Abbreviations: MG‐ADL, Myasthenia Gravis‐Activities of Daily Living; QMG, Quantitative Myasthenia Gravis.

^a^ Two MG‐ADL late responders had no follow‐up assessments following the timepoint at which they were registered as first responding because their first MG‐ADL response occurred at Week 130 of the open‐label extension.

^b^ One QMG early responder had no follow‐up assessments after becoming a QMG responder because the patient withdrew from the study after Week 12 of the open‐label extension.

Early responders as assessed by QMG scores also achieved a statistically significant improvement in the corresponding total score (LSM percentage change from baseline −20.8%; 95% CI: −26.2%, −15.5%) by Week 1 (Fig. 1). Late responders showed a steady improvement in the total score from Week 12, reaching statistical significance from Week 20 (LSM percentage change from baseline −13.5%; 95% CI: −24.0%, −3.0%) (Fig. 1). At Week 130, LSM percentage change from baseline was −40.8% (95% CI: −48.3%, −33.4%) and −55.5% (95% CI: −68.4%, −42.7%) for early and late QMG responders, respectively (Fig. 1). Nineteen (34.5%) of the 55 QMG early responders were classified as responders at all assessments including and following the timepoint at which the response was initially observed. The majority of early QMG responders (42/55; 76.4%) were classified as responders at >50% of timepoints including and following the first response timepoint. A QMG response was recorded at 72.2% (642/889) of assessments after the first response.

Of the 15 QMG late responders, one (6.7%) was classified as being a QMG responder at every timepoint including and following the initial response, while nine (60.0%) were classified as QMG responders at >50% of timepoints including and following the first response timepoint. A QMG response was recorded at 57.2% (83/145) of assessments after the first response. For the 49 early and late responders (71.0%) who did not maintain the 5‐point QMG response at every subsequent assessment, 12 patients (24.5%) maintained at least a 3‐point response (Table 3). Further breakdown of the data for patients maintaining a QMG response is shown in Table S1.

### Predictors of time to response

Patient demographics and baseline characteristics were evaluated to determine whether any predictors of time to response could be identified. The findings according to response classification for MG‐ADL and QMG responders are shown in Table S2. There were no differences in baseline characteristics between early and late MG‐ADL responders. Differences were seen between the early and late QMG responders: the mean duration of MG at baseline was longer (10.46 [95% CI: 8.34, 12.57] vs. 5.46 [95% CI: 3.97, 6.95] years); the mean baseline QMG score was greater (18.6 [95% CI: 17.1, 20.0] vs. 15.1 [95% CI: 12.9, 17.4]); and the mean baseline QMG bulbar‐component score was higher (1.1 [95% CI: 0.7, 1.6] vs. 0.6 [95% CI: 0.2, 1.0]) in the early responders (Table S2). No differences in the examined characteristics were seen between non‐responders and either early or late responders, with the exception of the mean baseline MG‐ADL limbs‐component score, which was higher in early responders than non‐responders (2.9 [95% CI: 2.6, 3.2] vs. 2.0 [95% CI: 1.3, 2.7]) and the mean baseline QMG limbs‐component score, which was also higher in early responders than non‐responders (11.4 [95% CI: 10.5, 12.3] vs. 8.5 [95% CI: 7.2, 9.7]). Of note, the baseline characteristics of the analysis population (*n* = 98) were similar to those of the REGAIN study population as a whole (*n* = 125), suggesting that the analysis population is representative of the study population.

There were more patients with prior exacerbations in the MG‐ADL early‐responder group (55/66 [83.3%]) than the late‐responder group (10/17 [58.8%]), and more patients with a thymectomy among non‐responders (11/15 [73.3%]) compared with early (38/66 [57.6%]) and late responders (9/17 [52.9%]). Among MG‐ADL non‐responders, 33.3% (5/15) had previously used four or more ISTs compared with 16.7% (11/66) and 17.6% (3/17) of early and late responders, respectively. No clear differences were seen for these variables among the QMG responder groups.

Evaluation of the number of assessments conducted in each of the response groups showed no differences between early versus late responders for either MG‐ADL or QMG. Non‐responders had fewer assessments than either the early or late responders for either measure; however, fewer assessments reflected the higher proportion of patients in the non‐responder group discontinuing from the study. With regard to response according to MG‐ADL score, of the 15 non‐responders, 10 (66.7%) discontinued the study (7/10 [70.0%] patient decision), while only 15/66 (22.7%) early responders and 2/17 (11.8%) late responders discontinued. For QMG response groups, 12/28 (42.9%) non‐responders discontinued the study (9/12 [75.0%] patient decision), while only 11/55 (20.0%) early responders and 4/15 (26.7%) late responders discontinued.

As the dose of concomitant ISTs could be modified at the investigator’s discretion during the OLE, we explored whether such changes could have contributed to the clinical responses. Changes in IST use before and after the first response for patients treated with eculizumab by responder category are shown in Table 4. Very few patients in any of the responder categories increased the daily dose of their IST or started a new IST before their first documented clinical response. Of the late responders, two patients (2/17 [11.8%] with a late MG‐ADL response; 1/15 [6.7%] with a late QMG response) achieved their first response just after receiving IVIg for clinical deterioration. None of the late responders received PLEX during the study before their first response.

**Table 4:** Changes in IST use before and after response for patients treated with eculizumab by responder category.

IST change	MG‐ADL				QMG			
	Early responders (*n* = 66)		Late responders (*n* = 17)		Early responders (*n* = 55)		Late responders (*n* = 15)	
	No. of events	Patients, *n* (%)	No. of events	Patients, *n* (%)	No. of events	Patients, *n* (%)	No. of events	Patients, *n* (%)
Started a new IST								
Total	50	24 (36.4)	9	6 (35.3)	34	17 (30.9)	18	8 (53.3)
Before the first response	1	1 (1.5)	0	0	0	0	3	1 (6.7)
On or after the first response	49	24 (36.4)	9	6 (35.3)	34	17 (30.9)	15	8 (53.3)
Increased daily dose of an IST								
Total	93	29 (43.9)	13	5 (29.4)	71	23 (41.8)	16	5 (33.3)
Before the first response	0	0	1	1 (5.9)	0	0	1	1 (6.7)
On or after the first response	93	29 (43.9)	12	5 (29.4)	71	23 (41.8)	15	4 (26.7)
Increased daily dose of >1 IST								
Total	0	0	2	2 (11.8)	2	2 (3.6)	0	0
Before the first response	0	0	0	0	0	0	0	0
On or after the first response	0	0	2	2 (11.8)	2	2 (3.6)	0	0

Abbreviations: IST, immunosuppressive therapy; MG‐ADL, Myasthenia Gravis‐Activities of Daily Living; QMG, Quantitative Myasthenia Gravis.

### Incidence of adverse events according to response

No clear differences were observed in the adverse‐event incidences for early and late responders using either the MG‐ADL or QMG response definitions. For MG‐ADL responders, 59.7 serious adverse events per 100 person‐years were observed for early responders and 45.9 per 100 person‐years for late responders. For QMG responders, 52.3 serious adverse events per 100 person‐years were observed for early responders and 62.8 per 100 person‐years for late responders.

## Discussion

The efficacy and tolerability of eculizumab in patients with anti‐AChR‐positive refractory gMG were demonstrated in the 26‐week REGAIN study and its OLE; however, patterns of response were not evaluated during the original analyses. In this retrospective analysis, responder data for patients enrolled in the REGAIN trial and its OLE were assessed to examine patterns of variability and to understand whether any factors could be identified that would allow the prediction of time to first response. Two measures were included—the MG‐ADL scale and the QMG instrument—with responders defined as those achieving a ≥3‐point or ≥5‐point reduction in their total score, respectively. Of note, these thresholds exceed the changes considered to be clinically meaningful, that is, a 2‐point reduction in the MG‐ADL score^20^ and a 3‐point reduction in the QMG total score,^21^ and are therefore more rigorous criteria.

The analysis of response patterns showed that while the majority of patients treated with eculizumab showed a response within the first 12 weeks of treatment, some took longer to respond, and a minority of patients did not achieve a response (as defined for the analysis) within the eculizumab treatment period across the REGAIN study and its OLE. Although some patients had received placebo in REGAIN, the potential impact of any placebo effect persisting into the OLE (and hence overlapping with the effects of eculizumab treatment) was avoided by omitting from the analysis patients with an MG‐ADL score of <6 at eculizumab initiation in the OLE. It is interesting to note that a higher proportion of non‐responders than early or late responders discontinued treatment (mostly “patient decision”) before study end. The discontinuations among non‐responders raise the question of whether a response might have been achieved had their treatment continued. Many patients maintained the stringently defined responses at all assessments following their initial responses, while others maintained responses that were above thresholds recognized as being clinically important. Variable responses and response durations are not unexpected, as gMG is characterized by fluctuating muscle weakness and fatigue. It is important to note, however, that a proportion of patients with previously refractory disease were able to maintain their response throughout the study period.

Complete terminal complement inhibition was documented in all patients regardless of their response status, which suggests that the degree of complement inhibition alone does not predict either response or time to response. Other studies have suggested that mechanisms other than complement‐mediated damage (e.g., steric hindrance by autoantibodies at the AChR; AChR turnover rate) also contribute to the pathophysiology of MG.^22^ To date, however, no clinical or preclinical studies have been conducted to investigate the relative contributions of these mechanisms to disease pathophysiology.

Interestingly, while there was a clear overlap between the MG‐ADL and QMG responder populations, there were also patients who responded according to one measure but not the other. The differences likely reflect the different nature of the measures, with the patient‐reported MG‐ADL score providing more subjective data than the physician‐reported QMG score. Analysis of the different responder groups using either measure showed no clear differences relating to disease severity, as reflected by the number of previous crises, prior exacerbations, history of thymectomy, or relating to previous IST use. Baseline differences between early versus late QMG responders were seen for the mean duration of MG, mean QMG score, and mean QMG bulbar‐component score. Baseline differences between early and non‐responders were seen for the mean QMG limbs‐component score and mean MG‐ADL limbs‐component score. However, we are not able to provide a physiological explanation for these observations and would urge caution when interpreting the results given the post hoc nature of the analysis and small patient numbers in some of the responder groups. Overall, the evaluation of baseline characteristics did not indicate specific profiles that would allow the prediction of patients who are likely to show an early versus late versus non‐response for either MG‐ADL or QMG.

When evaluating the findings, it is important to understand that physicians treating patients during the OLE of the REGAIN trial were able to modify the dose of concomitant ISTs. However, as reported elsewhere, more patients stopped or decreased the dose of an IST than started or increased the dose of an IST.^23^ In our analysis to explore whether IST changes could have contributed to patients’ responses, we observed that for the majority of responders, the initial response was not preceded by an increase in the daily dose of IST or initiation of a new IST, suggesting that these events were not important contributors to response. Notably, the majority of late first responses were not influenced by IST changes, nor by the administration of IVIg or PLEX.

Limitations of the analysis include the fact that MG‐ADL and QMG responders were defined by the achievement of a qualifying score at a single timepoint during eculizumab treatment. These responder definitions do not incorporate durability of response; this was, however, evaluated using data from follow‐up assessments throughout REGAIN and its OLE. In particular, one patient was defined as a QMG early responder at Week 12 of the OLE but had no subsequent follow‐up assessments because they withdrew from the study after this visit; the durability of their response, therefore, could not be evaluated. There was some variability in the number of assessments per patient over time and small patient numbers in some analysis groups. Regarding the former point, this was related to a reduction in the frequency of assessments conducted later in the trial. This could potentially have limited the ability of the analysis to capture within‐patient fluctuations when determining response stability over time. Additionally, the open‐label design and lack of concurrent placebo control in the trial extension could potentially have resulted in a higher responder rate over time, given that all patients were aware that they were receiving active treatment. Finally, while the lack of structured change in IST dosing during the OLE​—including the ability of investigators to increase background ISTs—may have indirectly contributed to an increase in response rate, further evaluations of the data (discussed above) showed that this limitation did not substantially affect the findings.

The findings from these analyses suggest that although most patients with refractory gMG will experience a clinical response (as assessed by MG‐ADL or QMG scores) by Week 12 of eculizumab treatment, first response can be observed with longer‐term treatment. Clinical practice may reflect the prescribing information, which states that response to eculizumab is usually achieved within 12 weeks; however, the current evidence suggests that this timeframe may need to be re‐appraised. Given the small number of patients in the analysis, it is not yet possible to determine how long beyond 12 weeks a patient should be treated before a decision is made to switch treatment, if an adequate response is not achieved. Despite the fluctuating nature of the illness, a high proportion of patients with previously refractory gMG maintained a response to eculizumab throughout the study period. Evaluation of baseline characteristics did not indicate specific profiles that would allow the prediction of patients who are likely to show an early versus late response according to either their MG‐ADL or QMG scores.

## Conflict of Interest

J.F.H. Jr received research support from argenx SE, Alexion Pharmaceuticals, and UCB‐Ra; advisory‐board honoraria from Alexion Pharmaceuticals, argenx SE, UCB‐Ra, Regeneron Pharmaceuticals, and Viela Bio Inc; and owns stocks in Johnson & Johnson and Pfizer. C.K. has served on advisory boards or provided advisory input for Acceleron, Alexion, Alnylam, Akcea, argenx, Biogen, CSL, Sanofi Genzyme, and UCB‐Ra, and has received research grants from Acceleron, Alexion, Alnylam, Akcea, argenx, CSL, Sanofi Genzyme, and UCB‐Ra. M.Y. and F.L.O’B. are employed by and own stocks in Alexion Pharmaceuticals. T.M. has served on advisory boards for AbbVie, Alexion, argenx, Audentes Therapeutics, Momenta, Sanofi Genzyme, Sarepta, Spark Therapeutics, The Myositis Association, The Neuromuscular Disease Foundation, and UCB‐Ra. In relation to these activities, he has received travel subsidies and honoraria. He has also served on the speaker’s bureaus for Alexion and Sanofi Genzyme, and receives research funding from the Myositis Association, the Muscular Dystrophy Association, and the National Institutes for Health, and from the following sponsors: Acceleron, Alexion, argenx, Audentes Therapeutics, Momenta, Sanofi Genzyme, Spark Therapeutics, UCB‐Ra, and Valerion. He serves on a data safety monitoring board for Acceleron.

## Supporting information


**Figure S1**. Patient populations for REGAIN, the REGAIN OLE, and the response analysis.
**Table S1**. Proportion of assessments where response was observed.
**Table S2**. Baseline and disease characteristics of patients treated with eculizumab.
**Appendix S1**. List of REGAIN and extension study investigators and collaborators.

## References

[1] Hehir MK , Silvestri NJ . Generalized myasthenia gravis: classification, clinical presentation, natural history, and epidemiology. Neurol Clin 2018;36:253–260. 29655448 10.1016/j.ncl.2018.01.002

[2] Silvestri NJ , Wolfe GI . Treatment‐refractory myasthenia gravis. J Clin Neuromuscul Dis 2014;15:167–178. 24872217 10.1097/CND.0000000000000034

[3] Meriggioli MN , Sanders DB . Muscle autoantibodies in myasthenia gravis: beyond diagnosis? Expert Rev Clin Immunol 2012;8:427–438. 22882218 10.1586/eci.12.34 PMC3505488

[4] Biesecker G , Gomez CM . Inhibition of acute passive transfer experimental autoimmune myasthenia gravis with Fab antibody to complement C6. J Immunol 1989;142:2654–2659. 2703710

[5] Christadoss P . C5 gene influences the development of murine myasthenia gravis. J Immunol 1988;140:2589–2592. 3356901

[6] Piddlesden SJ , Jiang S , Levin JL , et al. Soluble complement receptor 1 (sCR1) protects against experimental autoimmune myasthenia gravis. J Neuroimmunol 1996;71:173–177. 8982117 10.1016/s0165-5728(96)00144-0

[7] Fumagalli G , Engel AG , Lindstrom J . Ultrastructural aspects of acetylcholine receptor turnover at the normal end‐plate and in autoimmune myasthenia gravis. J Neuropathol Exp Neurol 1982;41:567–579. 6982313 10.1097/00005072-198211000-00001

[8] Conti‐Tronconi B , Tzartos S , Lindstrom J . Monoclonal antibodies as probes of acetylcholine receptor structure. 2. Binding to native receptor. Biochemistry 1981;20:2181–2191. 6786327 10.1021/bi00511a017

[9] Nakano S , Engel AG . Myasthenia gravis: quantitative immunocytochemical analysis of inflammatory cells and detection of complement membrane attack complex at the end‐plate in 30 patients. Neurology 1993;43:1167–1172. 8170563 10.1212/wnl.43.6.1167

[10] Sahashi K , Engel AG , Lambert EH , Howard FM Jr . Ultrastructural localization of the terminal and lytic ninth complement component (C9) at the motor end‐plate in myasthenia gravis. J Neuropathol Exp Neurol 1980;39:160–172. 7373347 10.1097/00005072-198003000-00005

[11] Mantegazza R , Antozzi C . When myasthenia gravis is deemed refractory: clinical signposts and treatment strategies. Ther Adv Neurol Disord 2018;11:1756285617749134. 29403543 10.1177/1756285617749134 PMC5791553

[12] Suh J , Goldstein JM , Nowak RJ . Clinical characteristics of refractory myasthenia gravis patients. Yale J Biol Med 2013;86:255–260. 23766745 PMC3670444

[13] Schneider‐Gold C , Hagenacker T , Melzer N , Ruck T . Understanding the burden of refractory myasthenia gravis. Ther Adv Neurol Disord 2019;12:1756286419832242. 30854027 10.1177/1756286419832242 PMC6399761

[14] Thomas TC , Rollins SA , Rother RP , et al. Inhibition of complement activity by humanized anti‐C5 antibody and single‐chain Fv. Mol Immunol 1996;33:1389–1401. 9171898 10.1016/s0161-5890(96)00078-8

[15] Howard Jr JF , Utsugisawa K , Benatar M , et al. Safety and efficacy of eculizumab in anti‐acetylcholine receptor antibody‐positive refractory generalised myasthenia gravis (REGAIN): a phase 3, randomised, double‐blind, placebo‐controlled, multicentre study. Lancet Neurol 2017;16:976–986. 29066163 10.1016/S1474-4422(17)30369-1

[16] Muppidi S , Utsugisawa K , Benatar M , et al. Long‐term safety and efficacy of eculizumab in generalized myasthenia gravis. Muscle Nerve 2019;60:14–24. 30767274 10.1002/mus.26447 PMC6619057

[17] Wolfe GI , Herbelin L , Nations SP , et al. Myasthenia gravis activities of daily living profile. Neurology 1999;52(7):1487. 10227640 10.1212/wnl.52.7.1487

[18] Barohn RJ , McIntire D , Herbelin L , et al. Reliability testing of the quantitative myasthenia gravis score. Ann NY Acad Sci 1998;841:769–772. 9668327 10.1111/j.1749-6632.1998.tb11015.x

[19] Jaretzki A , Barohn RJ , Ernstoff RM , et al. Myasthenia gravis: recommendations for clinical research standards. Task Force of the Medical Scientific Advisory Board of the Myasthenia Gravis Foundation of America. Neurology 2000;55:16–23. 10891897 10.1212/wnl.55.1.16

[20] Muppidi S . The myasthenia gravis‐specific activities of daily living profile. Ann NY Acad Sci 2012;1274:114–119. 23252905 10.1111/j.1749-6632.2012.06817.x

[21] Katzberg HD , Barnett C , Merkies IS , Bril V . Minimal clinically important difference in myasthenia gravis: outcomes from a randomized trial. Muscle Nerve 2014;49:661–665. 24810970 10.1002/mus.23988

[22] Koneczny I , Herbst R . Myasthenia gravis: pathogenic effects of autoantibodies on neuromuscular architecture. Cells 2019;8:671. 10.3390/cells8070671 PMC6678492 31269763

[23] Nowak RJ , Muppidi S , Beydoun SR , et al. Changes in concomitant immunosuppressive therapy use during a Phase 3 open‐label study of eculizumab in adults with generalized myasthenia gravis: an interim analysis. Neurology 2019;92(15 Supplement):P5.2–080. 10.3389/fneur.2020.556104 PMC7732596 33329303

